# Molecular features of lipoprotein CD0873: A potential vaccine against the human pathogen *Clostridioides difficile*

**DOI:** 10.1074/jbc.RA119.010120

**Published:** 2019-08-16

**Authors:** William J. Bradshaw, Jean-François Bruxelle, Andrea Kovacs-Simon, Nicholas J. Harmer, Claire Janoir, Severine Péchiné, K. Ravi Acharya, Stephen L. Michell

**Affiliations:** ‡ Department of Biology and Biochemistry, University of Bath, Claverton Down, Bath BA2 7AY, United Kingdom; § EA4043 Unité Bactéries Pathogènes et Santé (UBaPS), University Paris-Sud, Université Paris-Saclay, Châtenay-Malabry Cedex, France; ¶ College of Life and Environmental Sciences, University of Exeter, Exeter EX4 4QD, United Kingdom; ‖ Living Systems Institute, University of Exeter, Exeter EX4 4QD, United Kingdom

**Keywords:** protein structure, ABC transporter, vaccine, crystallography, microbiology, Clostridioides difficile, tyrosine metabolism

## Abstract

*Clostridioides difficile* is the primary cause of antibiotic-associated diarrhea and colitis, a healthcare-associated intestinal disease resulting in a significant fatality rate. Colonization of the gut is critical for *C. difficile* pathogenesis. The bacterial molecules essential for efficient colonization therefore offer great potential as vaccine candidates. Here we present findings demonstrating that the *C. difficile* immunogenic lipoprotein CD0873 plays a critical role in pathogen success *in vivo*. We found that in a dixenic colonization model, a CD0873-positive strain of *C. difficile* significantly outcompeted a CD0873-negative strain. Immunization of mice with recombinant CD0873 prevented long-term gut colonization and was correlated with a strong secretory IgA immune response. We further present high-resolution crystal structures of CD0873, at 1.35–2.50 Å resolutions, offering a first view of the ligand-binding pocket of CD0873 and provide evidence that this lipoprotein adhesin is part of a tyrosine import system, an amino acid key in *C. difficile* infection. These findings suggest that CD0873 could serve as an effective component in a vaccine against *C. difficile*.

## Introduction

In recent years, standard methods of treatment have become increasingly ineffective against antibiotic-associated diarrhea caused by *Clostridioides difficile*. Together with the emergence of epidemic and hypervirulent lineages, this has resulted in *C. difficile* infection (CDI)^6^ becoming an ever-growing burden on healthcare systems (1, 2). The economics of *C. difficile* vaccines have been modeled, demonstrating that a vaccine against *C. difficile* is economically viable and urgently required (3, 4). It has been proposed that a *C. difficile* vaccine should provide coverage against several pathogenic strains, prevent gastrointestinal colonization, or block cellular toxicity by secreted toxins (5). The most advanced vaccines trialed to date have focused predominantly on the *C. difficile* toxins alone, with some still the focus of clinical trials, whereas others having been withdrawn (5, 6). Many have suggested the development of vaccines that target the initial stages of CDI, such as colonization of the gut via adhesion to host cells, as a complementary strategy for new vaccines (7).

Several *C. difficile* surface molecules have been investigated as putative adhesion and colonization factors (8). These include, but are not limited to, members of the family of cell wall proteins (Cwp), the S-layer proteins (SLP), microbial surface components recognizing adhesive matrix molecules (MSCRAMMs) including fibronectin-binding protein (Fbp68/FbpA) and collagen-binding protein (CbpA) (9). Other proteins reported to have a role in *C. difficile* adherence are components of the flagellar apparatus, although these have been shown to function in a strain-dependent manner (10). The *C. difficile* antigen CD0873 is annotated as a substrate-binding protein component (SBP) of an ATP-binding cassette (ABC) transporter (11) and is an immunoreactive protein in human infection (12). We have previously shown, using both genetic and cellular approaches, that CD0873 is a surface-exposed lipoprotein and an adhesin of *C. difficile* (13).

Here we used a competitive murine model to demonstrate that a CD0873-deficient strain of *C. difficile* shows a long-term decrease in colonization fitness. We show that purified CD0873 can protect against long-term persistence in a conventional murine active immunization model, with a corresponding specific adaptive immune response to CD0873. We present three high-resolution structures of CD0873, which possesses a typical Class I SBP fold: a near-atomic resolution closed, ligand-bound structure, an open, ligand-bound structure, and an open, ligand-free structure. The structural and biochemical information reported in this study demonstrates that tyrosine is the ligand of CD0873. Given the importance of tyrosine metabolism in *C. difficile* persistence, through 4-methylphenol (*para*-cresol) production, these data provides essential information on this phenomenon (14, 15). To the best of our knowledge, we present the first crystal structures of a known Class I SBP adhesin. This report provides detailed characterization of a *C. difficile* protein, CD0873, which should be considered as a component of future vaccines to prevent *C. difficile* colonization.

## Results

### WT C. difficile outcompetes a CD0873 mutant in a dixenic murine model of colonization

**Figure 1.**
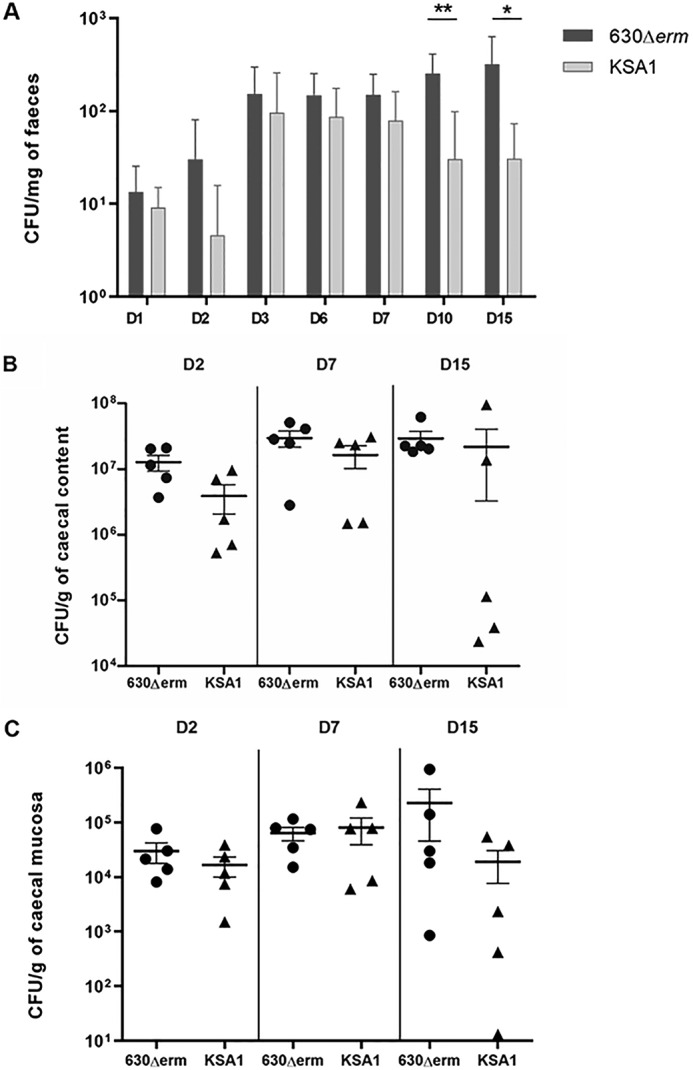
**Evaluation of intestinal colonization by *C. difficile* in the competitive dixenic mice infected by both 630Δ*erm* and KSA1 *C. difficile* strains, with equivalent inoculum.** *A,* mean of *C. difficile* vegetative cells in mouse feces at D1, D2, D3, D6, D7, D10, and D15 630Δ*erm* (*dark gray*) and KSA1 (*light gray*). *B,* mean of *C. difficile* vegetative cells in caecal contents of mice sacrificed on D2, D7, and D15 for the 630Δ*erm* group (*circle*) and KSA1 (*triangle*). *C,* mean of adherent *C. difficile* vegetative cells on caecal mucosa of mice sacrificed on D2, D7, and D15. Data and *error bars* are the mean ± S.E. calculated on counts obtained from mice per group (*n* ≥ 5). The nonparametric Mann-Whitney *U* test was used to compare data between groups; *, *p* < 0.05; **, *p* < 0.01.

It has previously been shown that the lipoprotein CD0873 facilitates adherence of *C. difficile* to human enterocytes (13). We therefore hypothesized that CD0873 may confer a fitness advantage to *C. difficile* in a dixenic murine model of colonization. To test this hypothesis, germ-free mice were co-challenged with wildtype (WT, 630Δ*erm*) and CD0873-inactivated *C. difficile*, KO strain (KSA1). Initially, both strains exhibited similar levels of colonization as observed by the bacteria shed in the feces at D(day)1 post-challenge (Fig. 1*A*). Bacterial fitness of the WT strain was significantly higher than the mutant KSA1 strain at D10 (*p* = 0.0043) and D15 (*p* = 0.025) after challenge, showing a higher level of bacterial shedding in feces (Fig. 1*A*). Study of the caecal content showed a decrease of the KSA1 strain at D15 compared with the WT 630Δ*erm* (Fig. 1*B*). The level of mucosa-associated bacteria was analyzed to assess the role of the lipoprotein CD0873 *in vivo* adhesion of *C. difficile* to gut mucosa. Although nonsignificant, at D15, a partial decrease of mucosa-associated KSA1 was observed compared with the WT strain (Fig. 1*C*). Taken together, these results indicate that, in this *in vivo* model, WT *C. difficile* outcompetes a CD0873 insertional mutant strain, suggesting that the lipoprotein CD0873 has a role in *C. difficile* gut colonization.

### Inactivation of the lipoprotein CD0873 reduces in vivo colonization by C. difficile in a conventional murine model of infection

**Figure 2.**
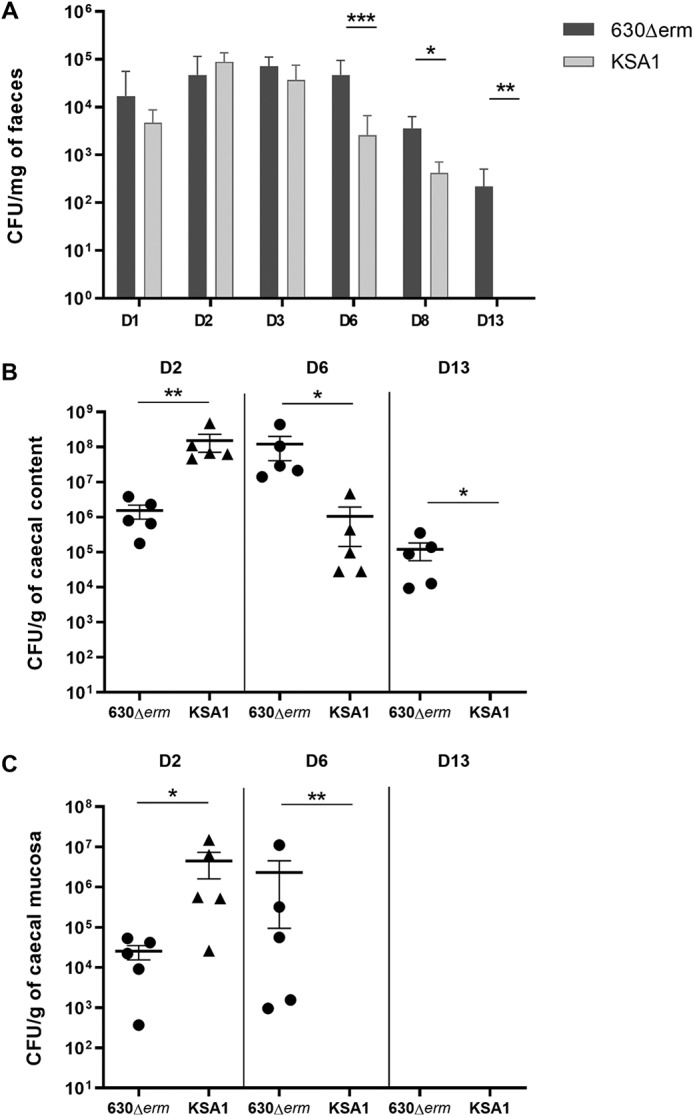
**Evaluation of intestinal colonization by *C. difficile* in conventional mice infected by 630Δ*erm* or KSA1 *C. difficile* strains.** *A,* mean of *C. difficile* vegetative cells in mouse feces at D1, D2, D3, D6, D7, D10, and D15 for the 2 groups 630Δ*erm* (*dark gray*) and KSA1 (*light gray*). *B,* mean of *C. difficile* vegetative cells in caecal contents of mice sacrificed on D2, D7, and D15 for the 630Δ*erm* group (*circle*) and KSA1 (*triangle*). *C,* mean of adherent *C. difficile* vegetative cells on caecal mucosa of mice sacrificed on D2, D7, and D15. Data and *error bars* are the mean ± S.E. calculated on counts obtained from mice per group (*n* = 5). The nonparametric Mann-Whitney *U* test was used to compare data between groups; *, *p* < 0.05; **, *p* < 0.01; ***, *p* < 0,001.

To further investigate the role of the lipoprotein CD0873 in CDI *in vivo*, the gut colonization ability of the CD0873 mutant strain of *C. difficile*, KSA1, was analyzed relative to that of its parental WT strain 630Δ*erm* in conventional mice that have a complex (although dysbiotic) gut microbiota. This previously developed reference mouse model of colonization mimics *C. difficile* infection associated with antibiotic treatment (16). Two groups of 15 mice were orally challenged with either *C. difficile* 630Δ*erm* or KSA1. Most of the infected mice developed diarrhea by D2 after challenge. However, fecal shedding of bacteria was significantly lower at D6 (*p* < 0.001), D8 (*p* = 0.012), and D13 (*p* = 0.0075), from mice challenged with KSA1 compared with mice challenged with 630Δ*erm*. This shows there is a significant and lasting decrease in colonization by the CD0873 mutant compared with the WT strain (Fig. 2*A*). Colonization of the gut by KSA1 was below the limit of detection at D13, whereas the WT remained detectable. These results were mirrored with the observation of a significantly lower level of KSA1 in the caecal content compared with that of the WT strain at D6 (*p* = 0.012) and D13 (*p* = 0.012) post-challenge (Fig. 2*B*). Surprisingly, at D2 after challenge, the level of luminal WT *C. difficile* was significantly lower than that of the CD0873 mutant KSA1 (*p* = 0.0079) (Fig. 2*B*), whereas no such difference was seen in the feces. Similar to the results reported from the dixenic model above, KSA1 appears to be cleared from the caecum at later stages of infection in this conventional model.

Gut colonization by the CD0873 mutant (KSA1) and WT strains was further investigated by measuring the numbers of bacteria associated with the caecal mucosa. Similar to the luminal analysis, at D2 after challenge a higher level of *C. difficile* KSA1 was associated with the caecum than the WT strain 630Δ*erm* (*p* = 0.032). However, this level decreased and at D6 the CD0873 mutant was significantly less associated to the mucosa than the WT strain (*p* = 0.0079) (Fig. 2*C*). Finally, at D13, the level of mucosa-associated bacteria was under the limit of detection in both groups. These results demonstrate that the CD0873 mutant exhibits a reduction in gut colonization in a mouse model of infection supporting the hypothesis that the lipoprotein CD0873 is a new colonization factor of *C. difficile*.

### Immunization with recombinant CD0873 protects mice from colonization by C. difficile and elicits a strong secretory IgA response

**Figure 3.**
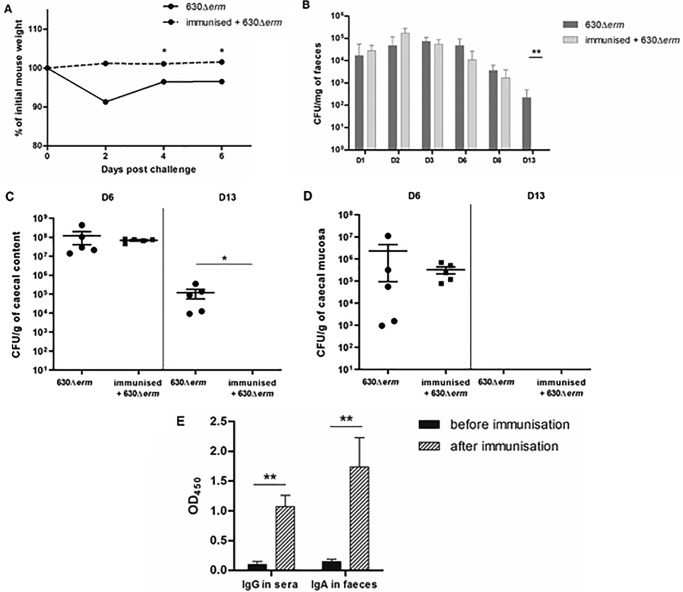
**Effects of CD0873 immunization on *C. difficile* intestinal colonization in mice.** *A,* survey of mouse weight expressed as mean of percentage of initial mouse weight for nonimmunized mouse group (630Δ*erm*) and immunized mouse group (immunized + 630Δ*erm*). *B,* mean of *C. difficile* vegetative cells in mouse feces at D1, D2, D3, D6, D8, and D13 for the immunized group (immunized + 630Δe*rm*) (*light gray*) and the nonimmunized control group (630Δ*erm*) (*dark gray*). *C,* mean *of C. difficile* vegetative cells in caecal contents of mice sacrificed on D6 and D13 for the immunized group (immunized + 630Δ*erm*) (*circle*) and the nonimmunized control group (630Δ*erm*) (*squares*). *D,* mean of adherent *C. difficile* vegetative cells on caecal mucosa of mice sacrificed on D6 and D13 for the immunized group (immunized + 630Δ*erm*) (*circle*) and the nonimmunized control group (630Δ*erm*) (*squares*). Data for the unimmunized mice in *B–D* is for comparison and represented from the data in Fig. 2. *E,* levels of specific Igs directed to CD0873 in mouse sera (IgG) or mouse feces (IgA) before (*black*) and after immunization (*gray*). *A–D*, the nonparametric Mann-Whitney *U* test was used to compare data between groups. *E,* data were analyzed by a paired *t* test. Normality was verified by the Shapiro-Wilk test and Kolmogorov-Smirnov test. Data and *error bars* are the mean ± S.E. calculated on counts obtained from mice per group (*n* = 5). *, *p* < 0.05; **, *p* < 0.01.

Given the importance of the lipoprotein CD0873 in *C. difficile* gut colonization, we assessed CD0873 as a vaccine candidate. Mice were intraperitoneally (IP) immunized with recombinant CD0873 formulated with Freund's complete and incomplete adjuvant, followed by oral challenge with WT *C. difficile* 630Δ*erm*. Gut colonization was compared with that in nonimmunized mice infected with the same WT strain. Surprisingly, vaccinated mice did not develop diarrhea after challenge contrary to the control. This was associated with a lower clinical score, meaning a lower weight loss and a better overall activity than nonvaccinated mice (Fig. 3*A*). This apparent protection was associated with a late decrease, significant at D13 after challenge, of *C. difficile* gut colonization in vaccinated mice compared with nonvaccinated mice, as shown in fecal shedding (*p* = 0.0075) (Fig. 3*B*) and luminal bacteria in caecum (*p* = 0.015) (Fig. 3*C*). At D13, the level of *C. difficile* associated with the caecum was under the limit of detection for both groups (Fig. 3*D*). As expected, the reduction in late stage colonization in CD0873-vaccinated mice was correlated with a significant production, after immunization, of anti-CD0873 IgG (*p* = 0.0019) in the serum and IgA in the intestine (*p* = 0.0042). These specific antibody levels were over 10-fold higher than in the nonimmunized group (Fig. 3*E*). Conjoined, these results further validate the lipoprotein CD0873 as a colonization factor of *C. difficile*. The data demonstrate that elicitation of a specific systemic and mucosal immune response after vaccination with recombinant CD0873 can protect against *C. difficile* morbidity in mice.

### The molecular structure of CD0873

**Figure 4.**
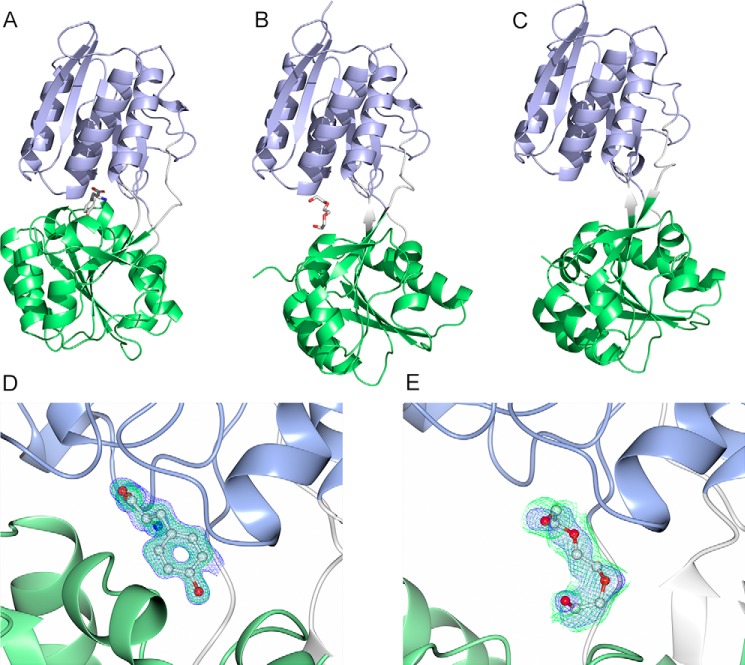
**The molecular structure of CD0873 and ligand electron density.** *A,* closed, ligand-bound conformation. *B,* open, ligand-bound conformation. *C,* open, ligand-free conformation. Domain 1 is shown in *blue*, the hinge region in *gray*, and domain 2 in *green*. Each domain has an α-β-α sandwich-fold. The closed conformation binds a tyrosine molecule at the interface between the two domains, which is likely to be the physiological ligand, whereas the open, ligand-bound conformation binds a PEG molecule, which will be a crystallographic artifact. *D,* tyrosine density for the closed, ligand-bound structure. The density is from the round of refinement immediately after molecular replacement. *E,* polder map of PGE density for the open, ligand-bound structure. Both images show 2*F_o_* − *F_c_* in *blue* at 1.5σ and *F_o_* − *F_c_* in *green* at 3σ.

Considering the role of CD0873 in *in vitro C. difficile* adherence (13) and in *in vivo* colonization, it was of interest to determine the structure of this protein to provide further molecular insight into its biological function. The structure of CD0873 was determined in three conditions, providing different information on ligand-binding. The closed, ligand-bound structure was determined to a resolution of 1.35 Å; the semi-open, ligand-bound structure was determined to 1.80 Å; and the open, ligand-free structure was determined to 2.50 Å (Fig. 4, *A–C*). Crystallographic and refinement statistics are given in Table 1. The recombinant plasmid, pNIC_KSA1, coded for Ser-25 to Glu-340 as the signal peptide of CD0873 is predicted to be cleaved before Cys-24 in the native form (resulting in the mature lipoprotein) (13), with Glu-43 to Gln-339 visible in the structures. CD0873 assumes a typical Class I (17) or Cluster B (18) SBP-fold. The protein possesses two domains, each consisting of an α-β-α sandwich joined by three loops that form a hinge. The ligand-binding site is formed by the interface between the two domains.

**Table 1:** **Crystallographic and refinement statistics**

Crystallographic statistics	Closed, ligand-bound	Open, ligand-bound	Open, ligand-free
Space group	C2	P2_1_2_1_2_1_	P2_1_2_1_2_1_
Unit cell dimensions (Å, °)	131.1, 42.4, 56.2	38.0, 57.5, 148.5	40.4, 62.5, 146.1
	90, 94.8, 90	90, 90, 90	90, 90, 90
Resolution	[55.98–7.27]*^a^*	[74.26–9.00]	[146.10–8.67]
	(1.37–1.35)	(1.84–1.80)	(2.61–2.50)
*R*_merge_	[0.133] 0.297 (6.455)	[0.085] 0.237 (1.483)	[0.055] 0.158 (0.673)
*R*_meas_	[0.138] 0.309 (6.880)	[0.089] 0.250 (1.561)	[0.061] 0.177 (0.796)
*R*_pim_	[0.037] 0.086 (2.319)	[0.028] 0.077 (0.483)	[0.026] 0.079 (0.344)
CC_1/2_	[0.997] 0.997 (0.327)	[0.993] 0.993 (0.721)	[1.000] 0.998 (0.782)
〈*I*/σ*I*〉	[30.2] 9.7 (1.7)	[16.4] 7.0 (1.8)	[17.7] 6.2 (1.9)
Total number of reflections	[11,357] 1,693,479 (58,056)	[4,916] 615,303 (35,222)	[2,640] 101,849 (9,836)
Number of unique reflections	[481] 67,816 (3,424)	[322] 31,122 (1,758)	[370] 12,793 (1,285)
Multiplicity	[23.6] 25.0 (17.0)	[15.3] 19.8 (20.0)	[7.1] 8.0 (7.7)
Completeness (%)	[99.8] 99.9 (98.8)	[99.9] 99.9 (98.3)	[96.1] 95.9 (80.2)
Refinement statistics			
*R*_work_/*R*_free_	0.149/0.186	0.204/0.239	0.251/0.310
RMSDs			
Bond lengths (Å)	0.016	0.012	0.009
Bond angles (°)	1.802	1.557	1.280
Ramachandran statistics (%)			
Favored	97.4	97.6	93.2
Allowed	2.3	2.0	6.2
Outliers	0.3	0.4	0.7
Average B-factors (Å^2^)			
Protein	18.6	30.5	68.2
Active site ligand	11.1	60.1	NA*^b^*
Other ligands	38.5	55.5	NA
Water	32.7	36.6	42.7
Number of atoms			
Protein	2269	2234	2200
Active site ligand	13	10	NA
Other ligands	23	31	NA
Water	307	143	12
PDB code	6HNI	6HNJ	6HNK

*^a^* Inner shell statistics are given in square brackets, outer shell statistics are given in round brackets.

*^b^* NA, not applicable.

**Figure 5.**
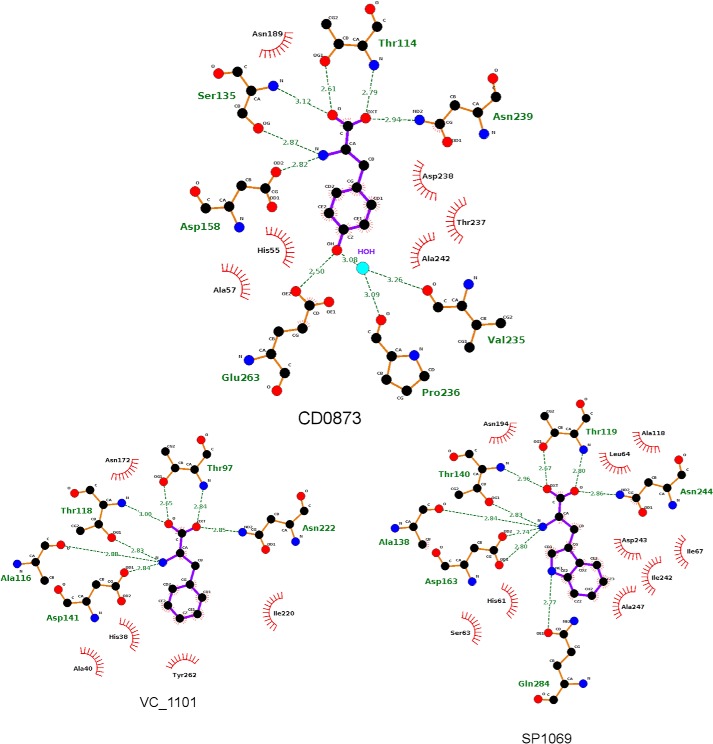
**Ligand binding of CD0873, VC_1101, and SP1069.** The method of ligand binding is largely conserved between the three proteins with variations in two residues that appear to control substrate selectivity. VC_1101, which binds phenylalanine, can be considered the “default.” Ala-246 in VC_1101 and Ala-268 in SP1069 are replaced by Glu-263 in CD0873, which forms a hydrogen bond to the hydroxyl of tyrosine, which is also stabilized by a hydrogen bond to a water, which is, in turn, stabilized by Val-235 and Pro-236. Tyr-279 in CD0873 and Tyr-262 in VC_1101 are replaced by Gln-284 in SP1069. The smaller side chain reduces steric hindrance, allowing tryptophan to bind, which is stabilized by a hydrogen bond to the side chain carbonyl of the glutamine. Residues shown as a ball and stick models interact with the ligand through hydrogen bonds, shown in *green*. Residues shown as *red lines* interact through van der Waals forces.

The closed structure has a tyrosine molecule (retained during purification) bound (Fig. 4, *A* and *D*). The same pocket has a polyethylene glycol (PEG) molecule bound (acquired from the crystallization medium, Fig. 4, *B* and *E*) in the semi-open structure. The open structure does not have any ligand bound (Fig. 4*C*). These high resolution structures provide a first “glimpse” of the physiological ligand-binding pocket of CD0873. The DALI server (19) identified two proteins with similar structures: VC_1101 from *Vibrio cholerae* (Z = 42.0, id = 38%, PDB code 3LKV) and SP1069 from *Streptococcus pneumoniae* (Z = 41.3, id = 36%, PDB code 3LFT). CD0873 superposes on VC_1101 and SP1069 with RMSDs of 0.85 and 1.18 Å, respectively. Notably, all three structures have an aromatic amino acid bound. CD0873 has a tyrosine molecule bound, whereas VC_1101 has phenylalanine bound and SP1069 has tryptophan bound. Comparison of the structures shows that the proteins share a common mechanism of binding to the amino and acid groups, but show sequence differences that account for their side chain preferences (Fig. 5).

### Differential scanning fluorimetry

**Figure 6.**
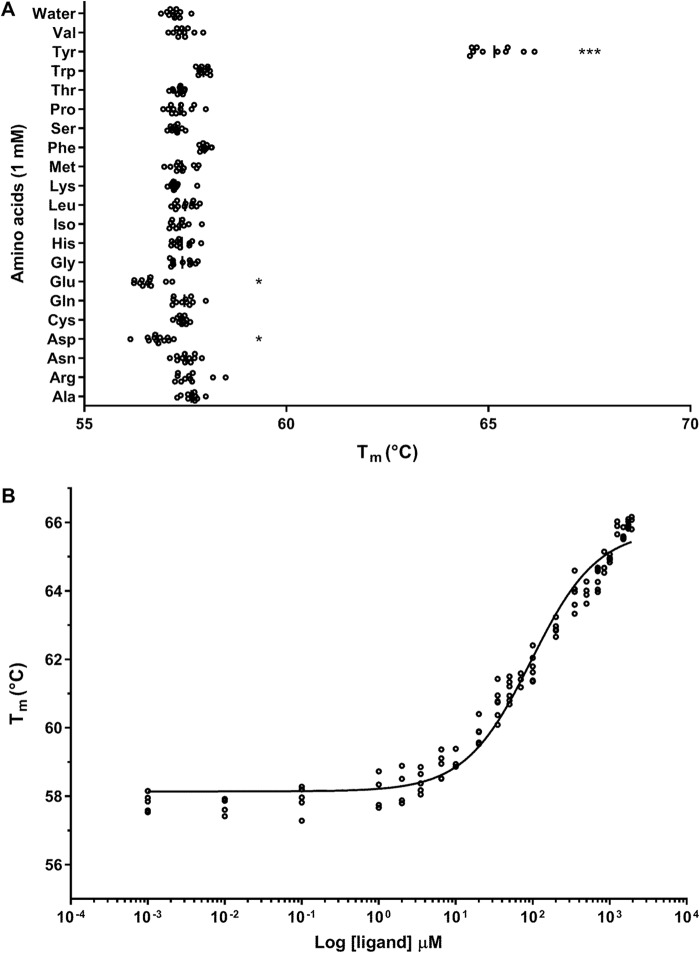
**Characterization of the CD0873 protein in the presence of amino acids determined by thermal shift assay.** *A,* stability of CD0873 in the presence of 20 amino acids. Increased melting temperature (*T_m_*) of CD0873 (***, *p* < 0.0001, ANOVA with Tukey's post hoc test) in the presence of tyrosine suggests interaction of CD0873 with tyrosine. Glutamic and aspartic acid both slightly destabilize the protein (*p* < 0.01). *B,* binding kinetics of CD0873 for tyrosine. CD0873 binds to tyrosine in a dose-dependent manner, showing a dissociation constant *K_D_* of 94 ± 7 μm. Experiments were performed in 6 replicates and the data are representative of two experiments.

The interaction of CD0873 with tyrosine was confirmed biophysically. The stability of the CD0873 protein was assessed in the presence of 20 natural amino acids using differential scanning fluorimetry (DSF) (20, 21). Refolded protein was used to ensure no amino acids remained bound from expression. Correct refolding of the protein was confirmed using CD (data not shown). DSF analysis strongly suggested, in agreement with the crystallization studies, that CD0873 specifically binds tyrosine (Fig. 6*A*; *p* < 0.0001). To further characterize the interaction, we determined the dose dependence of protein-ligand binding. These data (Fig. 6*B*) suggested a dissociation constant *K_D_* (the concentration where the protein is half-bound with ligand) of 94 ± 7 μm. These experiments have confirmed that the *C. difficile* adhesin CD0873 is a tyrosine substrate-binding protein of an ABC transporter system.

## Discussion

Here we show that CD0873 is an interesting vaccine candidate due to its key role in disease. The detailed structure of this antigen serves as a basis for a specific subunit vaccine for *C. difficile* and also deciphers why the antigen is required biologically by this pathogen. For the first time we have demonstrated the *in vivo* role of CD0873 in *C. difficile* intestinal colonization. In our dixenic mouse model, competition between the parental strain and the CD0873 mutant strain led to lower numbers of the mutant in the feces, caeca, and mucosa, relative to the parental WT strain. This persistence in the intestinal tract by the parental strain can thus be attributed to the expression of CD0873. This observation further supports previous findings of the up-regulation of CD0873 upon binding to Caco2 cells as well the direct adherence of CD0873 to enterocytes (13, 22). To reinforce these data, we used a conventional murine model in which we also observed a defect in long-term colonization for the CD0873-deficient *C. difficile* strain. This observation aligns with the previous reports that an isogenic CD0873 mutant of *C. difficile* shows dramatically reduced adherence to Caco-2 enterocytes *in vitro* (13). In our murine models, dysbiosis allows the WT *C. difficile* to proliferate and establish infection. Recent studies have shown that the specific ability of *C. difficile* to generate *p*-cresol, as the main fermentation product of tyrosine, is key to this pathogen's ability to cause disease. This is supported by the observation that a *C. difficile* mutant defective in metabolizing tyrosine to *p*-cresol shows a fitness disadvantage in a murine model of infection, a similar phenotype to that reported here for our CD0873 mutant (14, 15, 23). The bacteriostatic properties of *p*-cresol, which *C. difficile* itself can tolerate, provides these bacteria with a competitive advantage over other gut microflora, enabling them to proliferate and cause CDI (14, 15).

It is interesting to note that many clinical strains have a reduced ability to produce *p*-cresol and this has been correlated with a convergent loss of two genes from a neighboring ABC transporter, CD0876 and CD0877 (24). The solute binding protein CD0876 has 70% identity to CD0873 and due to minimal differences in residues in the present structures demonstrated to be involved in formation of the ligand-binding site, we propose CD0876 to also be a tyrosine transporter. However, CD0877, the ATP-binding protein of the CD0875-7 ABC transport system, has 88% identity to CD0874, and the former is also missing in these clinical isolates that exhibit reduced *p*-cresol production (24). It is therefore possible that CD0876 is also a tyrosine-binding protein and that the reduction in *p*-cresol observed in these clinical strains is due to deletion of the ATP-binding protein, CD0877. This possibility arises from the observation that the solute binding component of ABC- transport systems provide specificity and are subsequently able to form functional transport systems with reciprocal permeases and ATP-binding proteins (25). Therefore, inactivation of CD0873, resulting in a lack of tyrosine uptake, may lead to less *p*-cresol being produced by *C. difficile* and thus a faster restoration of normal intestinal microbiota of mice. In agreement with this hypothesis, our KSA1 strain was cleared more rapidly than a WT strain of *C. difficile* expressing CD0873 in our models of infection. This hypothesis also is supported by the recent observation that a *C. difficile* mutant defective in metabolizing tyrosine to *p*-cresol also shows a fitness disadvantage in a murine model of infection (15). The convergent loss of CD0876 and CD0877 from clinical isolates may arise from the high numbers of *C. difficile* present during infection, with an associated elevated level of *p*-cresol generating a selection pressure to lose one of the two tyrosine transport systems to stabilize the production of this bacteriostatic compound. CD0873 is retained over CD0876 potentially due to its presence providing an advantageous adherent phenotype although this role in pathogenesis is yet to be fully elucidated.

The structural and biophysical data presented here unequivocally demonstrate that tyrosine is the preferred ligand for CD0873. The binding sites of CD0873, VC_1101 (*V. cholerae*), and SP1069 (*S. pneumoniae*) are largely similar with important variations in two residues that are likely to affect the specificity of the three proteins (Fig. 5). The exquisite specificity of CD0873 for tyrosine is likely achieved through the tyrosine OH group. This is hydrogen bonded to the side chain of Glu-263, and to the main chain carbonyls of Val-235 and Pro-236 through a water molecule. In VC_1101 and SP1069, Glu-263 is replaced by an alanine: the lack of an appropriate hydrogen bond donor in these proteins explains their preference for more hydrophobic amino acids. Gln-284 in SP1069 aids binding of tryptophan through a hydrogen bond to the indole nitrogen; this glutamine is replaced by a tyrosine in CD0873 and VC_1101, which prevents binding of the larger tryptophan side chain through steric hindrance. When the proposed alternative tyrosine transporter, CD0876, is modeled against CD0873, an alanine at position 262 in CD0873 is replaced with a serine in CD0876. The hydroxyl group on this serine may affect the hydrogen bonding to the hydroxyl group of tyrosine although a structure of CD0876 would be required to confirm any effect on binding. As expected, the 18 residues at the N terminus of CD0873 were not ordered in the crystal structures. The fact that 18 N-terminal amino acids are not visible in any of the structures also aligns with previous conclusions that bacterial lipoproteins, and specifically SBPs, are connected to their membrane anchor via a disordered N-terminal linker (26, 27). These natively disordered juxtamembrane sequences encode information for the sorting of lipoproteins, and provide conformational flexibility to allow for correct positioning in the membrane. This is consistent with the role of CD0873 as an adhesin, allowing it to be presented outside the S-layer, thus facilitating its accessibility to antibodies, correlating with our previous observations that antibodies against CD0873 can inhibit binding of the bacteria to enterocytes (13). There are several proteins that have been determined to possess dual roles as a substrate-binding protein and an adhesin. PEB1a from *Campylobacter jejuni*, which binds glutamate and aspartate, possesses a protruding strand-turn-strand motif that was speculated to potentially be responsible for the adhesive role of the protein (28, 29). Lam29 from *Lactobacillus mucosae* appears to be responsible for the import of cysteine and is also capable of binding to human blood group antigens A and B (30). It may, therefore, be possible that adhesin-SBPs interact with glycans found on the surface of host cells, however, more work is required to identify a host receptor for CD0873.

Targeting colonization factors in a vaccine strategy against *C. difficile* aims to neutralize bacterial propagation in the gut, limiting the infection process and bacterial dissemination to the environment. To this end, several vaccine candidates have been tested in different models and via different routes of immunization (7). Here we evaluated the efficacy of an intraperitoneal immunization strategy using purified CD0873 protein, observing a significant decrease of intestinal colonization in the immunized mice compared with the nonimmunized mice. This was correlated with the production of specific sIgA and IgG to CD0873 in the immunized mice. Moreover, compared with the nonimmunized mice, the immunized mice presented a decrease in the severity of the infection characterized by a lack of weight loss and diarrhea. There is a precedent for using bacterial adhesins as vaccine candidates, with numerous bacterial adhesins also characterized as lipoproteins, similar to CD0873, including the adhesin PsaA, a solute-binding lipoprotein of the Mn^2+^ ABC transporter of *S. pneumoniae* (31–33). However, how many of these molecules function in a broader context of disease is often unclear. This study, through structural analysis, reveals a biochemical rationale for why CD0873 is required for colonization. The rate of progress into the design of more specific vaccines against bacterial pathogens is being increased through detailed structural knowledge of promising candidates (34–36). The work presented here not only demonstrates that CD0873 is a potential vaccine candidate for the prevention of colonization by *C. difficile* but also provides the structural information to be able to further develop improved subunit vaccines and aid in a deeper understanding of fundamental *C. difficile* biology.

## Experimental procedures

### Bacterial strains and growth conditions

*C. difficile* strains were grown at 37 °C in an anaerobic work station (Don Whitley, United Kingdom) in an atmosphere of 10% CO_2_, 10% H_2_, and 80% N_2_, using prereduced brain heart infusion broth or agar (Oxoid). The following *C. difficile* strains were used in this study: 630Δ*erm*, WT (37), and a CD0873 mutant, KSA1 (*C. difficile* 630Δ*erm*ΩCD0873(::*ermB*::317 318; Ery^r^)) ((13)). For molecular cloning *Escherichia coli* C43(DE3) was routinely cultured in lysogeny broth (LB) with shaking at 200 rpm or on LB agar at 37 °C. For protein expression studies *E. coli* cells were grown in 2× YT liquid medium. When appropriate, antibiotics were added at the following concentrations: kanamycin, 50 μg/ml; thiamphenicol, 15 μg/ml; and erythromycin, 5 μg/ml.

### Competitive colonization assay, dixenic mouse model

Twenty axenic C3H/HeJ female mice (6 weeks old) from INRA, Orléans, France, were infected with vegetative cells of WT *C. difficile* (630Δ*erm*, 5.7 × 10^5^ CFU/mouse) and a *C. difficile* CD0873 mutant strain, KSA1 (630Δ*erm*ΩCD0873, 4 × 10^5^ CFU/mouse). The gavage suspension was prepared from two overnight cultures. Vegetative forms were counted by microscopy with a Malassez unit. The fitness and development capacity of KSA1 was compared with that of the WT strain by monitoring each of the bacterial populations from the feces, caecum luminal contents, and washed caecal tissues throughout the trial. Feces were collected at D1, D2, D3, D6, D7, D10, and D15. In addition, at D2, D7, and D15 after challenge, five mice per group were sacrificed to monitor luminal bacteria and bacteria adhering to the cecum. Caeca were removed, caecal contents collected, and caeca were washed three times and all samples were weighed. Caecum-adherent bacterial counts were determined after mixing washed caecum in a PBS/Tween 80 (10%) solution with Ultra-Turrax (IKA) for 1 min on ice. Bacterial shedding in feces, luminal, and adherent bacterial counts were determined after successive dilutions of samples, in PBS, were cultured on BHI-horse blood 4% agar plates for 48 h at 37 °C in an anaerobic chamber. The CD0873 mutant strain, KSA1, numbers were detected after culture on BHI-horse blood 4% agar plates containing 4 μg/ml of erythromycin. Numbers of the 630Δ*erm* WT strain were determined after culture on BHI-horse blood 4% agar plates and subtraction of KSA1 numbers. Data are expressed in cfu/mg of feces and cfu/g of caecal sample. The limit of detection of *C. difficile* by this technique was 1 cfu/mg of feces or 10 cfu/g of caecal sample.

### Conventional mouse model of colonization

Two groups of 15 C57Bl/6 female mice (6 weeks old) from Charles River Laboratories (France) received an antibiotic treatment in drinking water for 7 days. The mixture of antibiotics was composed of kanamycin (0.4 mg/ml), gentamicin (0.035 mg/ml), colistin (850 U/ml), metronidazole (0.215 mg/ml), and vancomycin (0.045 mg/ml) ((16)). The concentration of the antimicrobial mixture was calculated based on the average weight of mice and their expected water consumption. On the third day of treatment a dose of clindamycin (10 mg/kg) was administered intraperitoneally. One day after the end of antibiotic treatment, both groups were orally challenged with the same amount of vegetative *C. difficile* cells. One group of mice was infected with the WT 630Δ*erm* strain (WT) and the second group with KSA1 (630Δ*erm*ΩCD0873 mutant strain).

At D1, D2, D3, D6, D8, and D13 post-challenge, feces were collected and bacterial fecal shedding was monitored. At D2, D6, and D13 post-challenge, five mice per group were sacrificed to monitor luminal bacteria and bacteria adhering to the caecum. Samples were analyzed as described previously as for the dixenic competition assay. *C. difficile* WT strain was detected on BHI-horse blood 4% agar plates and KSA1 mutant strain on BHI-horse blood 4%, erythromycin, 4 μg/ml, agar plates.

### Immunization assay

One group of 15 C57Bl/6 female mice (6 weeks old) received three times, every 15 days, one dose of recombinant CD0873 (50 μg/kg) in Freund's adjuvant (complete for the first immunization and incomplete for the second and third immunizations, Sigma) by intraperitoneal injection (purification of recombinant CD0873 protein detailed below). Before the first dose and 15 days after the last, blood and feces were sampled to analyze anti-CD0873 systemic IgG and local IgA responses, respectively. Blood was collected by submandibular bleeding under anesthesia (ketamine 1000, 100 mg/kg, Rompun 2%, 0.25 ml/kg). One fecal sample per mouse was suspended in 500 μl of PBS and protease inhibitor mixture (Roche Diagnostics). After centrifugation at 12,000 × *g* for 10 min at 4 °C, supernatants were used for anti-CD0873 IgA detection by ELISA.

Seven days after the last immunization, mice received an antibiotic treatment in drinking water for 7 days as described previously (16). Mice were then orally challenged with WT *C. difficile* (630Δ*erm*, 2.3 × 10^5^ cfu). At D1, D2, D3, D6, D8, and D13 post-challenge, feces were collected and bacterial fecal shedding was monitored. At D7 and D15 post-challenge, five mice per group were sacrificed to monitor luminal bacteria and bacteria adhering to the caecum. After challenge, weight loss was also monitored. Animals with a weight loss of more than 20% and/or a significant decrease of activity were euthanized to limit animal suffering. Results were compared with the nonimmunized group challenged with 630Δ*erm* from the colonization assay in the conventional mouse model. Data were represented to reduce the number of animals used in these studies.

### Detection of anti-CD0873 antibodies

Anti-CD0873 IgG and IgA were detected in serum and feces, respectively, by indirect ELISA as described previously (38). Briefly, 96-well microtiter plates (MaxiSorp, Nunc) were coated with 5 μg of recombinant purified CD0873 protein. Fecal supernatants were tested at a dilution of 1:2 and mouse sera at a dilution of 1:20. After washing, anti-CD0873 antibodies were detected by successive incubations for 30 min at 37 °C with a goat anti-mouse IgG or IgA conjugated to biotin (1:20,000 and 1:10,000 dilution, respectively; Sigma) and for 30 min at 37 °C with a streptavidin-horseradish peroxidase conjugate (1:5,000 dilution; Thermo Scientific). All samples were treated simultaneously and tested in duplicate to avoid inter-assay variation. Assays with antigen in the absence of sera served as negative controls.

### Statistical analysis

For non-normally distributed data from the *C. difficile* gut colonization analysis in dixenic and conventional mouse models, the Mann-Whitney *U* test was used. For antibody production evaluation, data were analyzed by a paired *t* test. Normality was verified by the Shapiro-Wilk test and Kolmogorov Smirnov test. A *p* value of less than 0.05 was considered to indicate statistical significance. Differential scanning fluorimetry results were analyzed using an ANOVA test with Tukey post hoc test using GraphPad version 8.2.0.

### Protein expression and purification

*E. coli* C43(DE3) cells harboring the pNIC_KSA1 vector (13) were grown in 2× YT media supplemented with 50 μg/ml of kanamycin. Cells were grown at 37 °C to an *A*_590_ of 0.5. Expression of the His-tagged recombinant protein was induced by addition of isopropyl 1-thio-β-d-galactopyranoside (0.5 mm) and the temperature reduced to 20 °C. Bacteria were harvested after 20 h, and suspended in 20 mm Tris-HCl (pH 8.0), and 0.5 m NaCl (Buffer A). The cells were lysed using a Soniprep 150 sonicator (MSE) with a medium probe. The lysate was clarified by centrifuging at 20,000 × *g* for 30 min at 4 °C. The supernatant was loaded on to a HisTrap Crude FF column (GE Healthcare). The column was washed with Buffer A supplemented with 25 mm imidazole-HCl (pH 8.0), and the protein eluted with buffer A supplemented with 250 mm imidazole-HCl (pH 8.0). His-tagged CD0873 was then loaded onto a HiLoad 16/600 pg Superdex 200 column (GE Healthcare) and eluted isocratically into 10 mm HEPES (pH 7.0) and 0.5 m NaCl (Buffer B). The chromatography steps were performed on an ÄKTAxpress chromatography system.

For preparation of tag-free CD0873, ∼25 mg of affinity purified protein was incubated at 4 °C for 43 h with 250 μg of TEV protease (His-tagged) (39). To remove residual undigested CD0873 and the TEV enzyme the sample was then loaded over a His GraviTrap column (GE Healthcare) and the flow through was retained. His tag cleavage was confirmed by SDS-PAGE.

For DSF and CD the untagged CD0873 protein was unfolded then refolded to remove any tyrosine bound during expression. Unfolding was performed by dialyzing the protein against 8 m urea. The denatured protein was refolded stepwise by dialysis with 6, 4, 2, and 1 m urea and finally Buffer C (50 mm borate-NaOH and 50 mm NaCl, pH 9.0). The refolded protein was loaded onto a HiLoad 16/600 pg Superdex 200 column (GE Healthcare) and eluted isocratically with Buffer C. The correctly refolded protein was concentrated to ∼1 mg/ml using a Vivaspin 6 centrifugal concentrator (MWCO 10,000 Da). For DSF experiments, the solvent Buffer C was replaced with Buffer B using a PD-10 desalting column (GE Healthcare).

### X-ray crystallographic studies

CD0873 was concentrated in a spin concentrator to a range of concentrations and screened against a wide range of molecular dimension crystallization conditions by sitting drop vapor diffusion. Crystals were observed from protein concentrated to 7.6 mg ml^−1^ mixed 1:1 (protein:condition) with Structure Screen 1&2 (SS1&2) condition G4 (0.1 m sodium HEPES, pH 7.5, 20% (w/v) PEG 10,000), 2.8 mg ml^−1^ mixed 2:1 with the BCS Screen condition A12 (0.1 m MES, pH 6.5, with 2% each of PEG 400, 500 MME, 600, 1000, 2000, 3350, 4000, 5000 MME, 6000, 8000 and 10,000), and 2.8 mg ml^−1^ mixed 1:1 with the BCS Screen condition C6 (0.2 m potassium chloride, 7.5% PEG 6000, 7.5% PEG 8,000, 7.5% PEG 10,000). The crystals were cryoprotected by addition of 1 μl of reservoir solution and data were collected from two crystals grown in SS1&2 G4 on beamline I04, a single crystal grown in BCS A12 on beamline I03 and a single crystal grown in BCS C6 on beamline I04-1 at Diamond Light Source, UK. The data were integrated with Dials (40) and scaled with Aimless (41).

The closed, ligand-bound structure (from SS1&2 G4 crystals) was solved by molecular replacement in Phaser (42) using VC_1101 (PDB 3LKV) as a model. The structure was refined and completed with Coot (43) and Refmac5 (44). Poor density was observed for Ser-328 to Asn-331 so this region was modeled with Rosetta (45). The modeled residues fit the density well both before and after final rounds of refinement. The two domains from the closed, ligand-bound structure were used separately in molecular replacement to solve the open, ligand-bound structure (BCS A12) and the open, ligand-free structure (BCS C6), which were refined and completed with Coot and Refmac5. All structures were validated with MolProbity (46).

### Differential scanning fluorometry

DSF assays were set up in a 384-well white quantitative PCR plates and sealed with optical tape (Applied Biosystems). Samples contained 0.1 mg/ml (2.95 μm) of CD0873, 10 mm HEPES (pH 7.0), 150 mm NaCl, and 5× SYPRO Orange dye in a final reaction volume of 10 μl. Amino acids (×10 concentrated stock solutions in water, except tyrosine, which was less concentrated due to poor solubility in water) were added to a final concentration of 1 mm for the initial screen, and between 1 nm and 1.93 mm for the dilution series of tyrosine. All samples were prepared in six replicates. The DSF reactions were performed using a QuantStudio 6 Flex Real-Time PCR System (Applied Biosystems). To generate the melting curves, samples were heated in a gradient from 25 to 99.9 °C (0.017 °C rise/s) and the fluorescence was measured at every 0.2 °C rise. The Boltzmann-derived melting temperature (*T_m_*) was determined using Protein Thermal Shift^TM^ Software version 1.3 (Applied Biosystems), and plots were produced using GraphPad Prism 8.2.0. The dissociation constant *K_D_* was determined using the equation,

(Eq. 1) $$T_{m}=T_{L}+\left[\left(T_{H}-T_{L}\right)\times \left(1-\frac{P-K_{D}-\left[Y\right]+\sqrt{{\left(P+\left[Y\right]+K_{D}\right)}^{2}-\left(4\times P\times \left[Y\right]\right)}}{2\times P}\right)\right]$$

where *K_D_* is the dissociation constant, and *P* the protein concentration (in the same units that were used for the ligand concentrations); and *T_H_* and *T_L_* are the melting temperatures at infinite ligand concentration and no ligand concentration, respectively (21). Protein concentration was treated as a constant, and *K_D_*, *T_H_*, and *T_L_* fitted to the data using Equation 1.

### Ethics statement

The protocols involving animals and their care were conducted in conformity with the institutional guidelines that are in compliance with national and international laws and policies. The protocol was approved by the Committee on the Ethics of Animal Experiments number 26 of the University of Paris-Sud and the French Minister of Research (2012-107). All efforts were made to minimize animal suffering.

## Author contributions

W. J. B., J.-F. B., A. K.-S., N. J. H., and S. P. data curation; W. J. B., J.-F. B., A. K.-S., N. J. H., C. J., S. P., K. R. A., and S. L. M. formal analysis; W. J. B. and K. R. A. validation; W. J. B., J.-F. B., A. K.-S., N. J. H., C. J., S. P., K. R. A., and S. L. M. methodology; W. J. B. and J.-F. B. writing-original draft; W. J. B., J.-F. B., A. K.-S., N. J. H., C. J., S. P., K. R. A., and S. L. M. writing-review and editing; C. J. and K. R. A. resources; C. J. investigation; C. J. and S. L. M. project administration; K. R. A. and S. L. M. supervision; S. L. M. conceptualization.

## Footnotes

6 The abbreviations used are:

CDI: *C. difficile* infection
SBP: substrate-binding protein
DSF: differential scanning fluorimetry
D: day
ANOVA: analysis of variance
TEV: tobacco etch virus.
